# The complete chloroplast genome sequence of a rambler rose, *Rosa wichuraiana* (Rosaceae)

**DOI:** 10.1080/23802359.2019.1700198

**Published:** 2019-12-12

**Authors:** Wei-Hua Cui, Mi-Cai Zhong, Xin-Yu Du, Xiao-Jian Qu, Xiao-Dong Jiang, Yi-Bo Sun, Dan Wang, Sui-Yun Chen, Jin-Yong Hu

**Affiliations:** a School of Life Science, Yunnan University, Kunming, Yunnan, China;; b CAS Key Laboratory for Plant Diversity and Biogeography of East Asia, Kunming Institute of Botany, Chinese Academy of Sciences, Kunming, Yunnan, China;; c Kunming College of Life Science, University of Chinese Academy of Sciences, Kunming, Yunnan, China;; d Biocontrol Engineering Research Center of Plant Disease and Pest, Yunnan University, Kunming, Yunnan, China;; e Biocontrol Engineering Research Center of Crop Disease and Pest, Yunnan University, Kunming, Yunnan, China;; f Germplasm Bank of Wild Species, Kunming Institute of Botany, Chinese Academy of Sciences, Kunming, Yunnan, China;; g Key Lab of Plant Stress Research, College of Life Sciences, Shandong Normal University, Jinan, Shandong, China

**Keywords:** *Rosa wichuraiana*, chloroplast genome, phylogenetic analysis

## Abstract

The rambler *Rosa wichuraiana* Crép. is an important founder species during modern rose domestication. However, the chloroplast genome (plastome) of this wild species remains unavailable. Here, we assembled the complete chloroplast genomes for two genotypes of *R. wichuraiana*. Both plastomes were typical quadripartite circular with 156,500/156,504 bp in length, comprising a large single-copy (LSC) region of 85,651/85,660 bp and a small single-copy (SSC) region of 18,751/18,744 bp, separated by two inverted repeat (IR) regions of 26,049/26,050 bp, respectively. Both plastomes encoded 113 unique genes, including 79 protein-coding genes, 30 tRNA genes, and 4 rRNA genes. Phylogenetic reconstruction with several rose plastomes revealed that both genotypes were sisters to a clade including *Rosa luciae*, *Rosa multiflora*, and *Rosa maximowicziana*.

*Rosa wichuraiana* Crép. is an important wild species that contributes the rambler habit to most of the climbing and groundcover cultivars of modern roses (Gerard 1897). This species was treated as a synonym of *Rosa luciae* var. *luciae* (section *Syntylae*), which has a native distribution in Southern-East of China, Japan, Korea and Philippines, but with no molecular evidence (Gu and Robertson 2003; Zhu et al. 2015). The genotype *R. wichuraiana* ‘Basye’s Thornless’ (designated as Rw01) conserved at Texas A&M University is especially valuable for its high resistance to black spot and powdery mildew, hardy, and prickleless traits (Byrne et al. 2007). It has been used as a model species for studying molecular genetic mechanisms underpinning the regulation of continuous flowering and other traits in woody plants (Semeniuk 1971; Shupert et al. 2007; Jones 2013; Dong et al. 2017; Li et al. 2018). In this study, the complete chloroplast genomes of Rw01 and a second *R. wichuraiana* genotype (Rw02) were assembled, and the phylogenetic position of *R. wichuraiana* was studied for the first time.

Here, fresh leaves of Rw01 were collected from plants cultivated in the glasshouse at the Flower Research Institute of Yunnan Academy of Agricultural Sciences (Kunming, China). The voucher specimen (1347952) was deposited in the Herbarium of Kunming Institute of Botany, CAS (KUN). Total DNA was extracted using a modified CTAB method and sequenced based on the Illumina pair-end technology (Biomarker Technologies Co., Ltd., Beijing, China) (Doyle and Doyle 1987; Li et al. 2019). The plastome was *de novo* assembled using SPAdes 3.12.0 (Bankevich et al. 2012), then built and annotated by Bandage 0.8.1 (Wick et al. 2015) and Geneious 9.1.4 (Biomatters Ltd., Auckland, New Zealand), using *Rosa chinensis* var. *spontanea* (NC038102) as reference. Raw reads of Rw02 were downloaded from NCBI (SRR6175519/SRR6175520) (Raymond et al. 2018), and the plastome of Rw02 was assembled and annotated as described before.

The plastomes of Rw01 (GenBank accession number: MN689790) and Rw02 (GenBank accession number: MN689791) were mostly identical (99.903%). They share a typical quadripartite circular with 156,500/156,504 bp in length, comprising a large single-copy (LSC) region of 85,651/85,660 bp and a small single-copy (SSC) region of 18,751/18,744 bp, separated by two inverted repeat (IR) regions of 26,049/26,050 bp, respectively. The overall GC content was ∼37.2%. The IR regions had a relatively higher GC content (42.7%) than LSC (39.7%) and SSC (31.4%) regions. Both plastomes encoded 113 unique genes, including 79 protein-coding genes, 30 tRNA genes, and 4 rRNA genes.

To determine the phylogenetic position of *R. wichuraiana*, the two newly assembled and 16 additional published Rosaceae plastomes were aligned using MAFFT (Katoh and Standley 2013). A maximum-likelihood tree was constructed using RAxML 8.2.11 (Stamatakis 2014) with GTRCAT substitution model and 1000 bootstrap replications. Species from *Rosa* section *Syntylae* formed a monophyletic clade with 100% bootstrap support (Figure 1). Rw01 and Rw02 formed a subclade, which was sister to the other subclade (100% support) including *R. luciae*, *Rosa multiflora*, and *Rosa maximowicziana*, a pattern contrast sharply with the previous report that *R. wichuraiana* was a subspecies of *R. luciae* (Gu and Robertson 2003). Our plastome sequences of *R. wichuraiana* genotypes provide new insights into evolutionary and genomic studies of Rosaceae.

**Figure 1.**
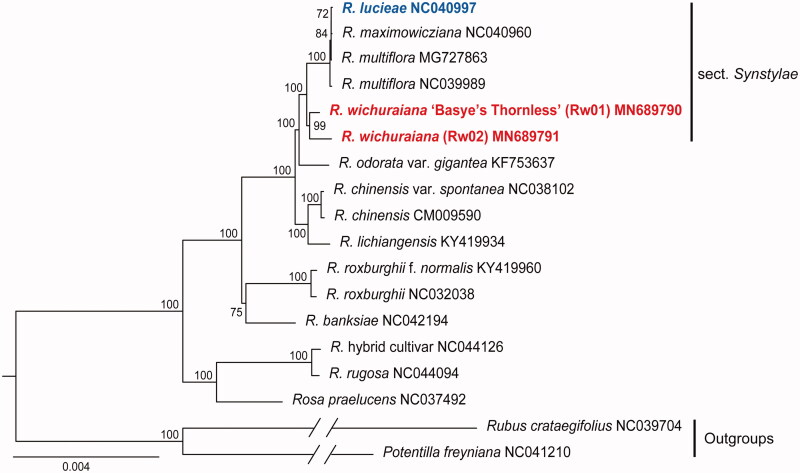
Phylogenetic tree based on 18 Rosaceae plastomes showing the relationships of *R. wichuraiana* (in red) with *R. luciae* (in blue). Bootstrap supports in percentage were shown along each branch.
